# Supplementary material for: Characterization of the Pristionchus pacificus “epigenetic toolkit” reveals the evolutionary loss of the histone methyltransferase complex PRC2
Source: Genetics. 2024 Mar 21;227(1):iyae041. doi: 10.1093/genetics/iyae041 (PMC11075575; doi:10.1093/genetics/iyae041)
Supplement: iyae041_Supplementary_Data [file iyae041_supplementary_data.zip › Supplemental_Material_GENETICS-2024-306912.pdf]

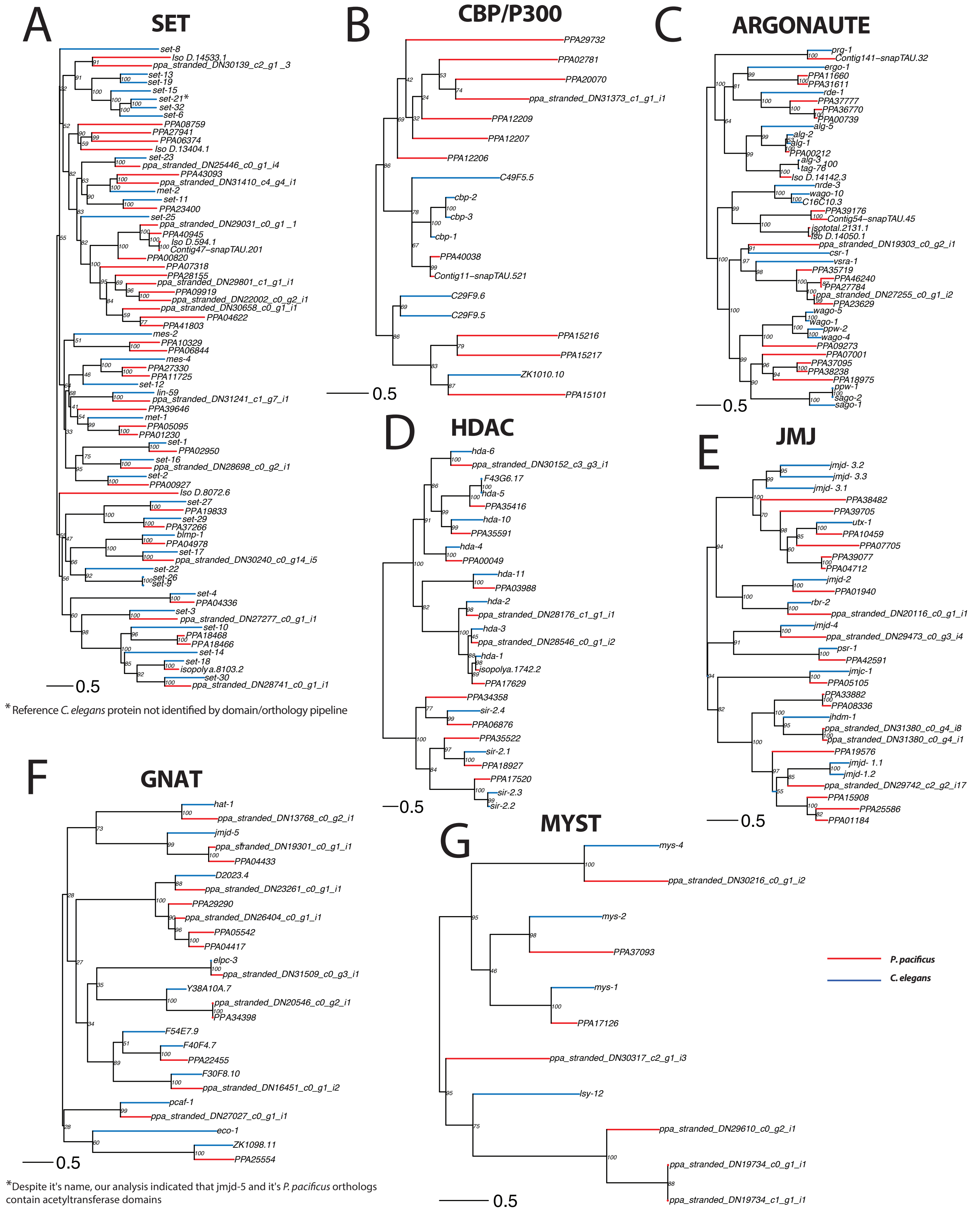

Figure S1: Phylogenies of putative *P. pacificus* and *C. elegans* epigenetic genes: Results for selected families demonstrate the occurence of one-to-one orthology and lineage-specific expansions. **A-G)** Mid-point rooted phylogenies of putative *P. pacificus* and *C. elegans* epigenetic genes generated using maximum likelihood from aligned protein sequences.

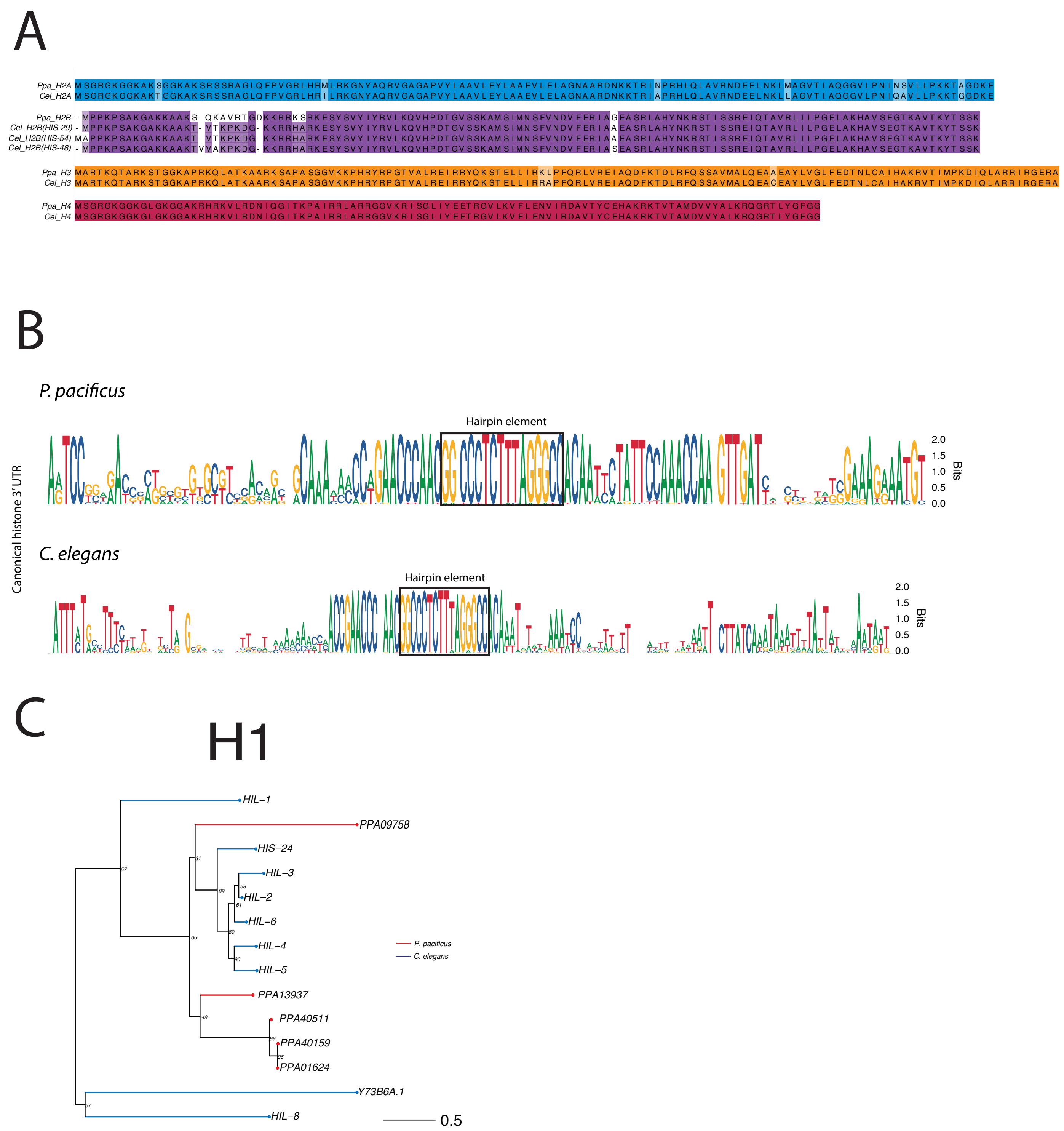

Figure S2: *P. pacificus* and *C. elegans* histone sequences. A) Amino acid alignments of canonical histone sequences. **B)** Nucleotide alignment of 3' UTR region for all one-exon histones. **C)** Unrooted H1 histone phylogeny generated using maximum likelihood from aligned histone amino acid sequences. Branch length reflects the average number of amino acid substitutions per site.

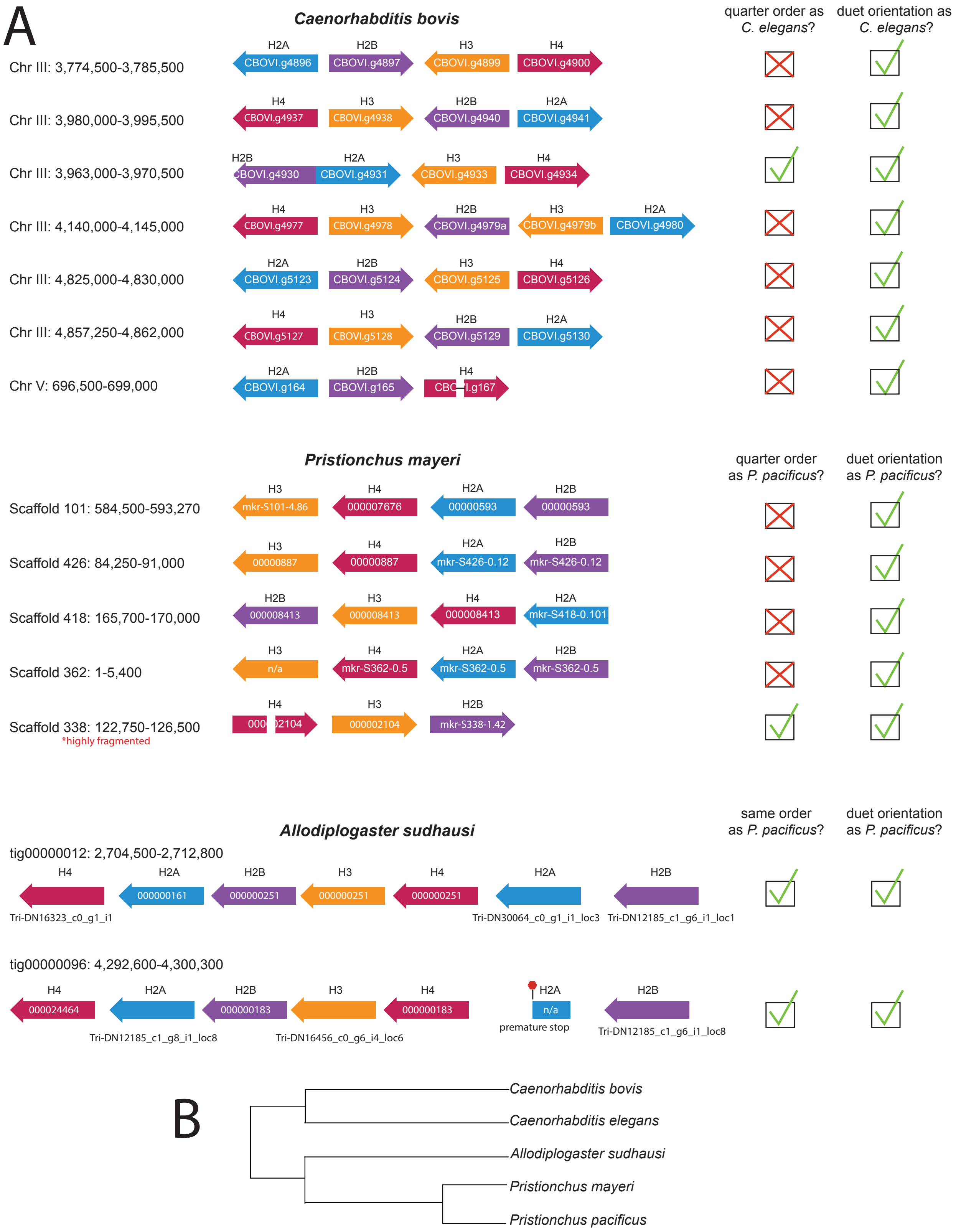

Figure S3: Histone-gene BLAST hits from related species shows deep histone cluster divergence. A) Histone clusters in *C. bovis*, *P. mayeri*, and *A. sudhausi* were identified by BLAST of H4 (as it is the most conserved histone), and manually examining for other histone genes in the vicinity. Note that the genomes of *P. mayeri* and *A. sudhausi* are assembled from short-read Illumina sequences and are of poorer overall quality, and thus we are likely missing several histone gene clusters. **B)** Representative phylogeny of *Caenorhabditis* and *Pristionchus* species.

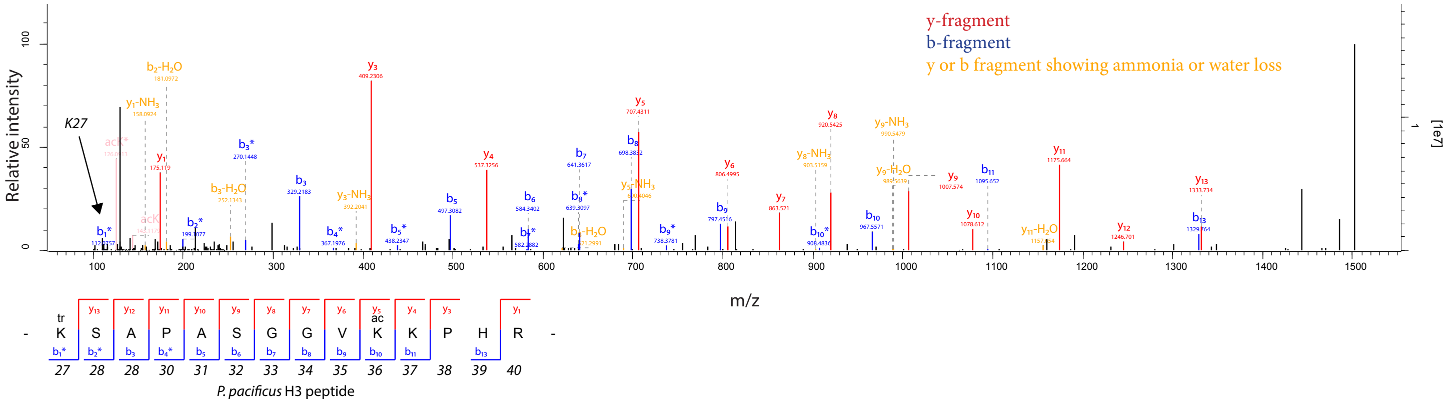

Figure S4: LC-MS/MS H3K27me3 spectra. LC-MS/MS spectra produced for *P. pacificus* H3 measuring mass to charge ratio vs. relative intensity. Arrow points to the peak corresponding to H3K27me3.

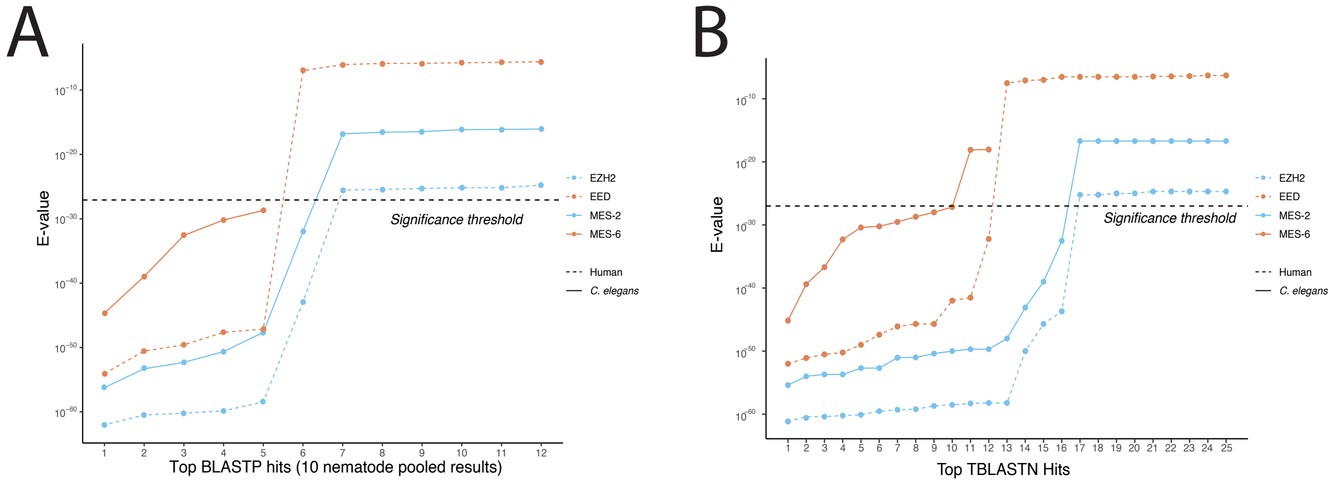

Figure S5: *Pristionchus* PRC2 BLAST threshold. A) Combined top 12 BLASTP hits of MES-2/EZH2 and MES-6/EED into each of the 10 diplogastrid nematodes which form a ladder-like phylogeny with *P. pacificus* (see phylogeny in Fig. 4E). **B)** Combined top 25 TBLASTN of MES-2/EZH2 and MES-6/EED into the transcriptome of each of the 10 diplogastrid nematodes which form a ladder-like phylogeny with *P. pacificus* (see phylogeny in Fig. 4E).

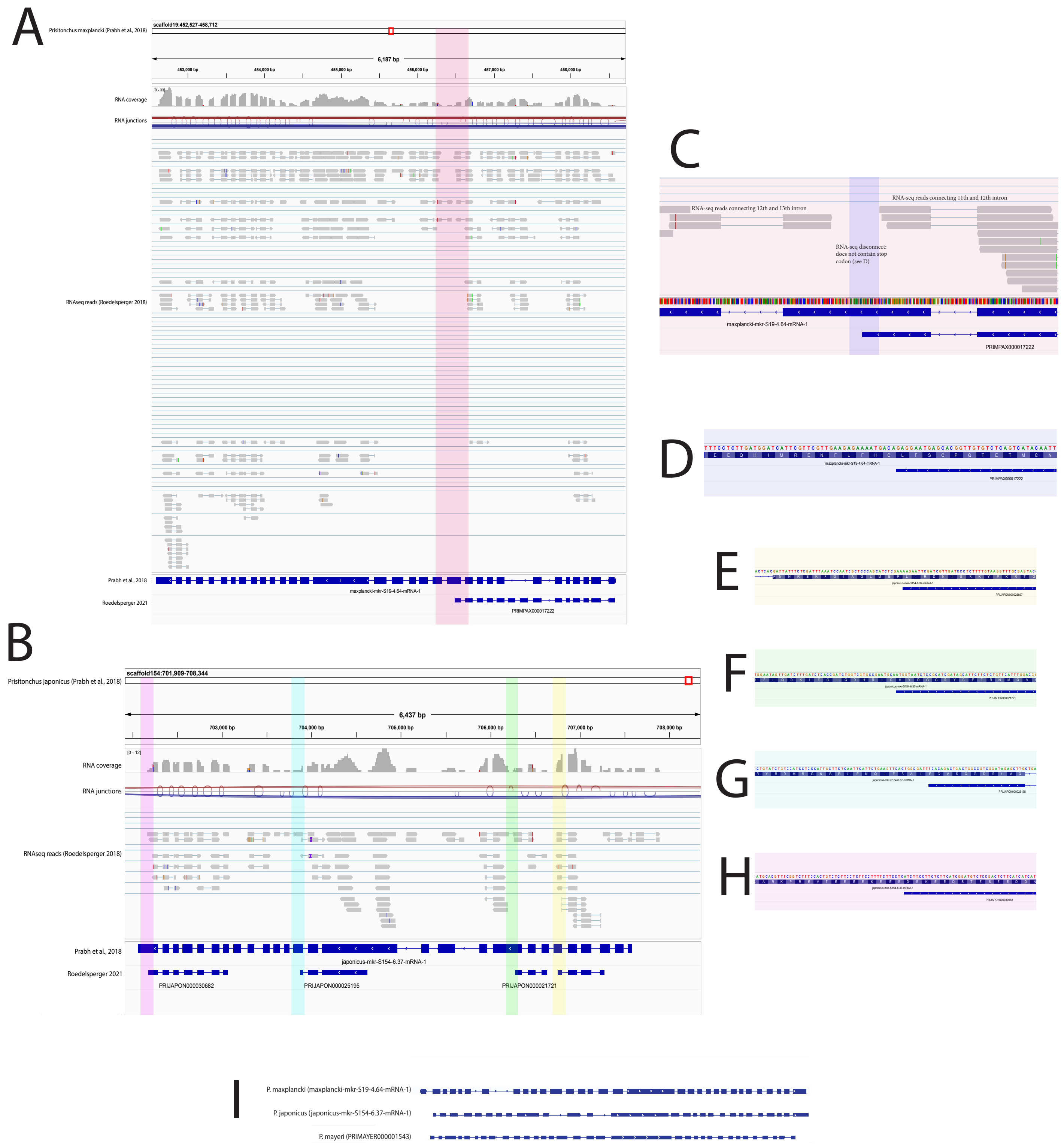

Figure S6: Rationale for gene re-annotation. The need for gene re-annotations was determined based on gene alignment with existing RNA sequencing reads and the identification of start/stop codons. Shown are representative examples where previous annotations (2018) of *mes-2* in *P. japonicus* and *P. maxplancki* were determined to be more accurate than the current annotations (2021). **A-B)** IGV image of gene annotations and RNA seq reads. **C)** Zoom of the pink region in A, RNA-seq reads spanning the 12th intron are shown. **D-G)** Zoom of colored regions in B, and blue region in C, showing the lack of stop codon in 2021 annotations. **I)** IGV image of EZH1/2 ortholog exon structure in *P. japonicus* (2018 annotation), *P. maxplancki* (2018 annotation), and *P. mayeri* (2021 annotation).

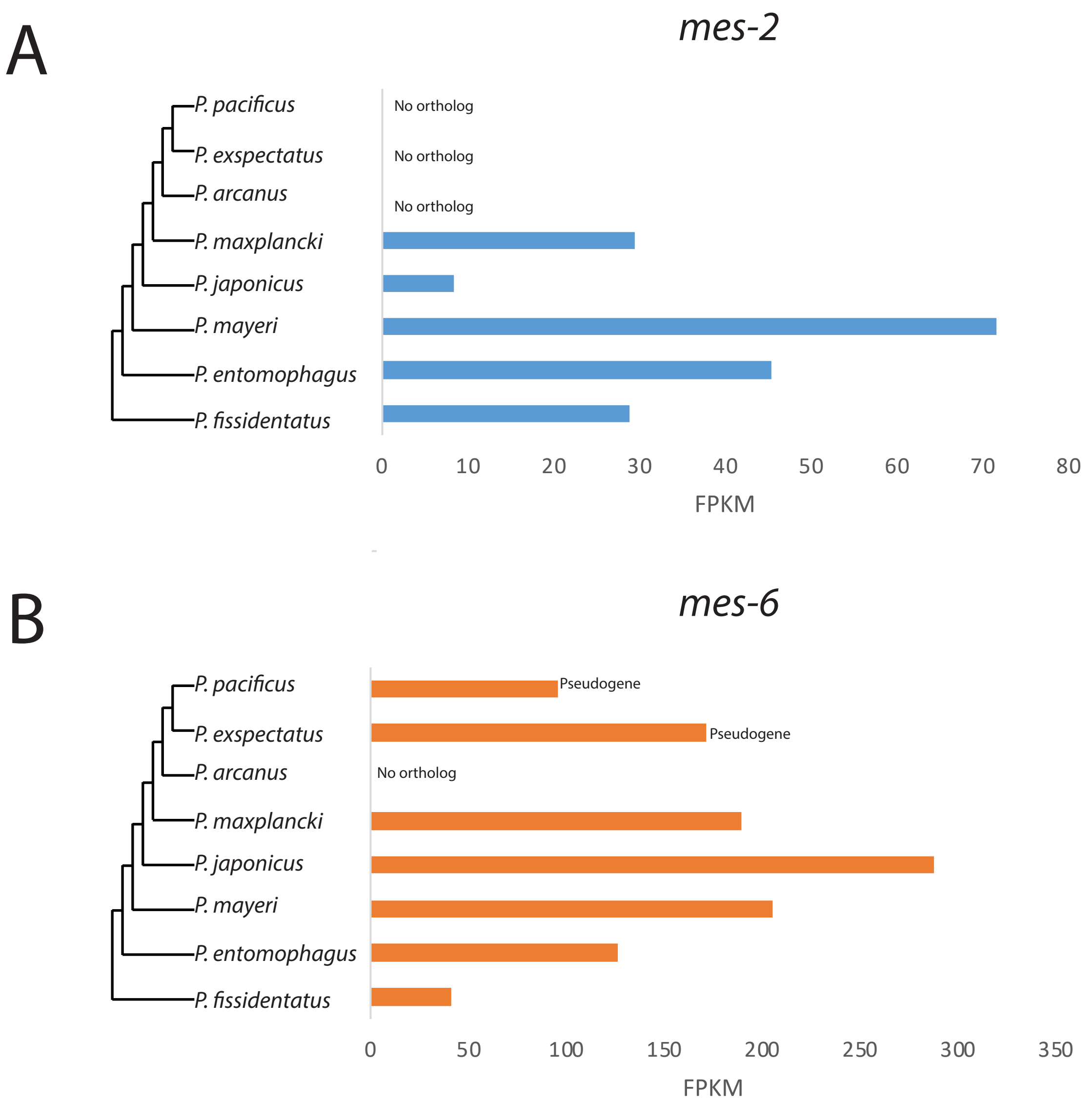

Figure S7: Gene expression of PRC2 components. A-C) Gene expression (FPKM) of *mes-2* and *mes-6* orthologs calculated from previous RNA sequencing reads from mixed-stage worms (Rödelsperger *et al*., 2018).

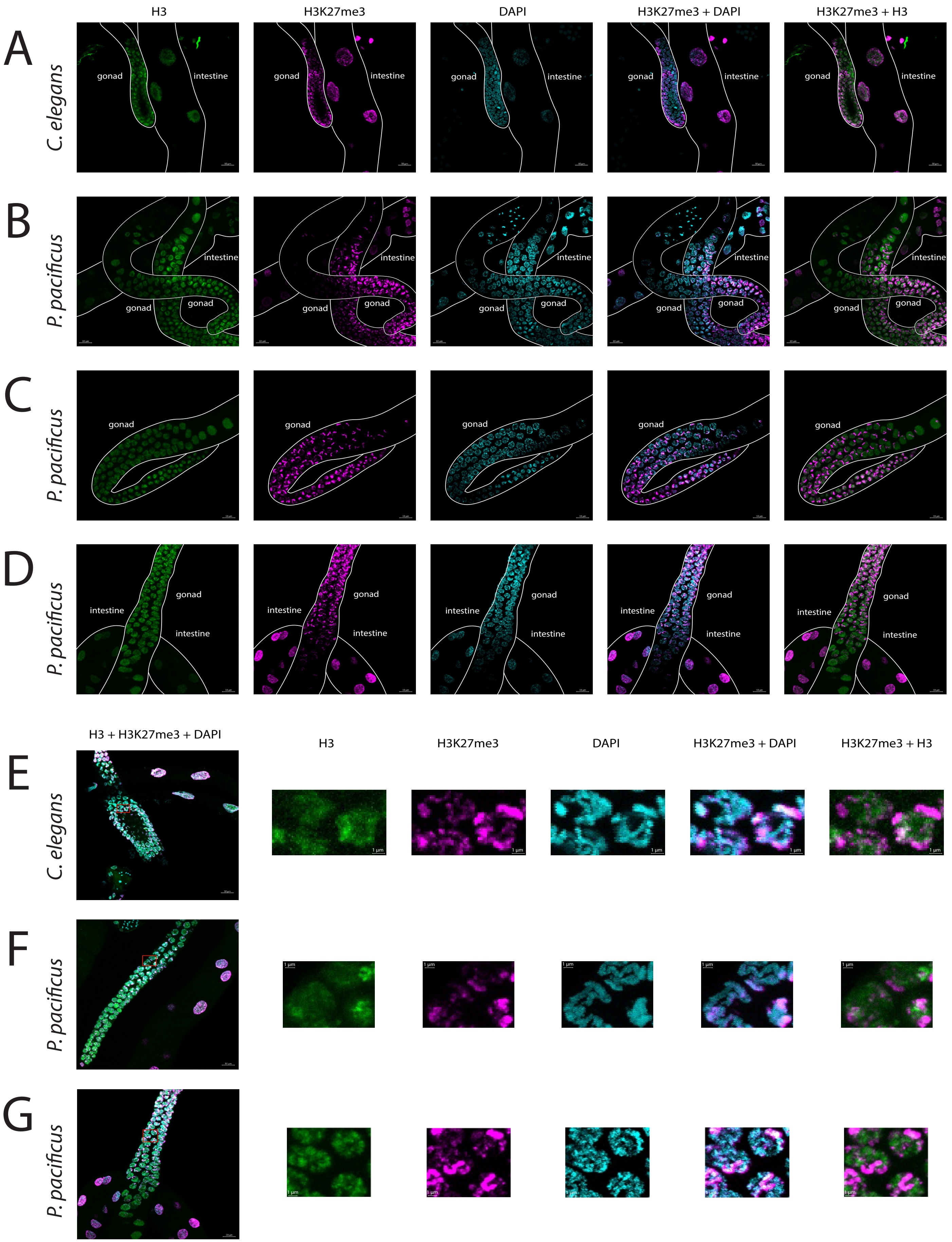

Figure S8: *P. pacificus* and *C. elegans* immunostaining. (A) Dissected *C. elegans* gonad and intestine **(B-D)** Dissected *P. pacificus* gonad and intestine **E-G)** Zoom of nuclei in *C. elegans* and *P. pacificus* gonad.

Species	Genome	Proteome	Transcriptome/RNA-seq/ ISO-seq
P. pacificus	'El Paco' assembly: Rödelsperger et al. (2017) Download: http://pristionchus.org/download/ PacBio reads: PRJEB20850	'El Paco' V3 annotations: Athanasouli et al., (2020) Download: http://pristionchus.org/download/	Werner et al., 2018 Download: http://pristionchus.org/download/
C. elegans	-	UniProt (Nov. 2022) ID: UP000001940	-
D. melanogaster	-	UniProt (Nov. 2022) ID: UP000000803	-
H. sapiens	-	UniProt (Nov. 2022) ID: UP000005640	-
A. thaliana	-	UniProt (Nov. 2022) ID: UP000006548	-
S. cerevisiae	-	UniProt (Nov. 2022) ID: UP000002311	-
S. pombe	-	UniProt (Nov. 2022) ID: UP000002485	-
P. exspectatus	Yoshida et al. (2023) Download: http://pristionchus.org/download/	Orthology pipeline: Rödelsperger et al., (2021) PRC2 analysis: Yoshida et al. (2023)	Rödelsperger et al. (2018) ENA Accession: PRJEB20959
P. mayeri	Prabh et al. (2018) Download: http://pristionchus.org/download/	Rödelsperger et al., (2021) Download: http://pristionchus.org/download/	Rödelsperger et al. (2018) ENA Accession: PRJEB20959
B. malayi	-	UniProt (Nov. 2022) ID: UP000006672	-
S. ratti	-	UniProt (Nov. 2022) ID: UP000035682	-
T. spiralis	-	UniProt (Nov. 2022) ID: UP000054776	-
A. pisum	-	UniProt (Nov. 2022) ID: UP000007819	-
A. mellifera	-	UniProt (Nov. 2022) ID: UP000005203	-
D. rerio	-	UniProt (Nov. 2022) ID: UP000000437	-
P. arcanus	Prabh et al. (2018) Download: http://pristionchus.org/download/	Rödelsperger et al., (2021) Download: http://pristionchus.org/download/	Rödelsperger et al. (2018) ENA Accession: PRJEB20959
P. maxplancki	Prabh et al. (2018) Download: http://pristionchus.org/download/	Rödelsperger et al., (2021) Download: http://pristionchus.org/download/	Rödelsperger et al. (2018) ENA Accession: PRJEB20959
P. japonicus	Prabh et al. (2018) Download: http://pristionchus.org/download/	Rödelsperger et al., (2021) Download: http://pristionchus.org/download/	Rödelsperger et al. (2018) ENA Accession: PRJEB20959
P. entomophagus	Prabh et al. (2018) Download: http://pristionchus.org/download/	Rödelsperger et al., (2021) Download: http://pristionchus.org/download/	Rödelsperger et al. (2018) ENA Accession: PRJEB20959
P. fissidentatus	Prabh et al. (2018) Download: http://pristionchus.org/download/	Rödelsperger et al., (2021) Download: http://pristionchus.org/download/	Rödelsperger et al. (2018) ENA Accession: PRJEB20959
M. japonica	Prabh et al. (2018) Download: http://pristionchus.org/download/	Rödelsperger et al., (2021)Download: http://pristion-chus.org/download/	Rödelsperger et al. (2018) ENA Accession: PRJEB20959
P. giblindavisi	Prabh et al. (2018) Download: http://pristionchus.org/download/	Rödelsperger et al., (2021) Download: http://pristion-chus.org/download/	Rödelsperger et al. (2018) ENA Accession: PRJEB20959
C. bovis	Stevens et al., (2020) ENA Accession: PRJEB34497	-	-
A. sudhausi	Wighard et al. (2022) Download: http://pristionchus.org/download/	-	-

Table S1: Genetic resources. Description of genetic resources used.
